# Effects of swimming exercise on nerve regeneration in a rat sciatic nerve transection model

**DOI:** 10.1051/bmdcn/2017070103

**Published:** 2017-03-03

**Authors:** Chien-Fu Liao, Tse-Yen Yang, Yung-Hsiang Chen, Chun-Hsu Yao, Tzong-Der Way, Yueh-Sheng Chen

**Affiliations:** 1 Department of Biological Science and Technology, China Medical University Taichung 404 Taiwan; 2 Department of Medical Research, China Medical University Hospital Taichung 404 Taiwan; 3 Graduate Institute of Integrated Medicine, China Medical University Taichung 404 Taiwan; 4 Department of Psychology, Asia University Wufeng District, Taichung 413 Taiwan; 5 Biomaterials Translational Research Center, China Medical University Hospital Taichung 404 Taiwan; 6 Department of Biomedical Informatics, Asia University Wufeng District, Taichung 413 Taiwan; 7 Lab of Biomaterials, School of Chinese Medicine, China Medical University Taichung 404 Taiwan

**Keywords:** Swimming exercise, Nerve regeneration, Muscular atrophy, Calcitonin gene-related peptide, Macrophage

## Abstract

Background: Swimming is commonly considered to be an efficient rehabilitation exercise to treat peripheral nerve injury. However, the most effective resistance level and exercise duration is still unclear. We investigated the effects and mechanisms of swimming at various exertion levels in a rat sciatic nerve transection model.

Methods: Sciatic nerve transection rats were randomized into the following four groups based on swimming duration (from the 7th day to the 28th day post-surgery): sedentary control group (SC), S10 group (10 min/3 times/week), S20 group (20 min/3 times/week), and S30 group (30 min/3 times/week) (n = 10 each). Axon regeneration, electrophysiological properties, muscular weights, macrophage infiltration, and nerve repair associated maker, calcitonin gene-related peptide (CGRP), were measured.

Results: Dramatic higher successful percentages of nerve regeneration across the 10-mm gaps in swimming groups compared to the SC group. Total area of nerve regeneration significantly improved in the S10 group; however, electrophysiological properties, muscular weights, and macrophage infiltration in the regenerated nerves of rats did not differ significantly between the various exercise groups. CGRP expression was significantly increased in the spinal cord of rats in the S20 group.

Conclusions: Our data indicated that CGRP-related axonal regeneration improved significantly with moderate swimming. These results should inspire new studies in physiotherapeutic practice for related human treatment.

**Abbreviations**
CGRP: calcitonin gene-related peptide
IL: interleukin
IR: immunoreactivity
MAPs: muscle action potentials
NCV: nerve conductive velocity
S: swam
SC: sedentary control

## 1. Introduction

Numerous therapeutic treatments for peripheral nerve regeneration, mostly physical interventions, have been studied. It has been reported that physical exercise, such as running on a treadmill or wheel, could promote peripheral nerve regeneration by increasing the number of regenerating nerve fibers, rate of axonal growth, and extent of muscle reinnervation [1, 2]. However, conflicting results on peripheral nerve regeneration have been reported; some studies have reported that both the forced exercises may cause detrimental effects, especially on the restoration of muscle function [3, 4]. Stress induced by forced physical training could be a factor that may impede functional recovery after nerve injury [5].

In comparison, only a few studies in the literature have examined the effect of swimming on the regeneration of injured peripheral nerves. It has been reported that animals that underwent swimming showed accelerated nerve regeneration compared to control animals with crush nerve injury by increasing the diameter of nerve fibers [6]. On the other hand, one study showed that intense swimming (two hours every day) could not enhance the restoration of muscle innervation following a crushed sciatic nerve [3]. Furthermore, another study showed that mice with sciatic nerve lesion that were made to swim over a period of 22 days showed no increased nerve regeneration compared to controls [7].

However, these prior studies only involved a small nerve defect [8]. Because of the inherent regenerative capacity of animals, the effects of swimming on regeneration may not be fully elucidated in cases of smaller injury. To provide essential evidence, we assessed the influence of swimming following a 10-mm sciatic nerve defect in rats, which was repaired with a silicone rubber nerve tube. After a four-week recovery period, calcitonin gene-related peptide (CGRP) in the lumbar spinal cord, macrophage infiltration in the distal nerve end, and morphology and electrophysiology of the regenerated nerve in the tube were performed with the aim of elucidating the mechanisms underlying the observed effects of swimming [9].

## 2. Experimental procedures

### 2.1. Experimental design and surgical procedures

**Fig. 1:**
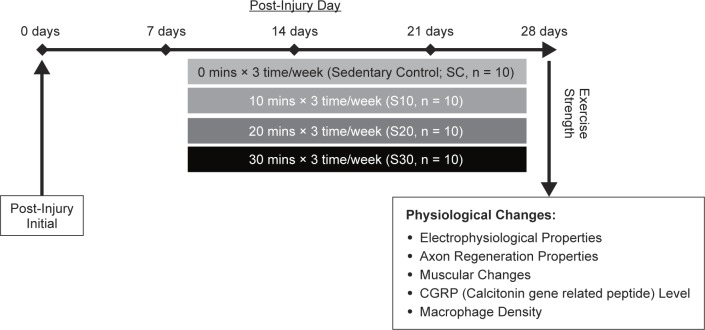
Experimental design. The swimming rehabilitated experimental processes were divided into 4 groups by exercise strength after seven days of nerve injury surgery. The subsequent rehabilitated swimming exercise was started from 7^th^day to 28^th^ day after post-injury surgery.

This study was approved by the ethical committee for animal experiments of the China Medical University, Taichung, Taiwan. The animals submitted to swimming underwent an adaptation period in a rectangular tank (67.0 × 46.5 × 41.0 cm) filled with water at 28°C. The adaptation phase lasted 10 minutes on the first day and was increased 10 minutes every other day over a week period to allow the animals to become familiarized with the swimming exercise. After the adaptation period, the animals were anesthetized with an inhalational technique (AErrane; Baxter, Deerfield, IL), whose right sciatic nerves were severed into proximal and distal segments. The proximal stump was then secured with a single 9-O nylon suture through the epineurium and the outer wall of a silicone rubber chamber (1.47 mm inner diameter and 1.96 mm outer diameter; Helix Medical, Inc., Carpinteria, CA). The distal stump was secured into the other end of the chamber. Both the proximal and the distal stumps were secured to a depth of 1 mm into the chamber, leaving a 10-mm gap between the stumps. The muscle and skin were closed. All animals were housed in temperature (22°C) and humidity (45%) controlled rooms with 12-hour light cycles. They had access to food and water ad libitum. The animals were then randomly divided into 4 groups: rats with sciatic repair and unexercised, sedentary control (SC, n = 10); rats in the other three groups swam 10 (S10, n = 10), 20 (S20, n = 10), and 30 (S30, n = 10) minutes, respectively, every other day beginning a week after the nerve repair. In short, the rehabilitated swimming exercise was started from 7^th^ day to 28^th^ day after post-injury surgery, and the study design was shown in Fig. 1.

### 2.2. Electrophysiological techniques

Four weeks after nerve repair, all animals were re-anesthetized and the sciatic nerve exposed. The nerve was given a supramaximal stimulus through a pair of needle electrodes placed directly on the sciatic nerve trunk, 5-mm proximal to the transection site. Latency, amplitude, and area of the evoked muscle action potentials (MAPs) were recorded from the gastrocnemius muscle with microneedle electrodes linked to a computer (Biopac Systems, Inc., Goleta, California). The latency was measured from stimulus to the takeoff of the first negative deflection. The amplitude and the area under the MAP curve from the baseline to the maximal negative peak were calculated. The MAP was then used to calculate the nerve conductive velocity (NCV), which was carried out by placing the recording electrodes in the gastrocnemius muscles and stimulating the sciatic nerve proximally and distally to the silicone rubber conduit. The NCV was then calculated by dividing the distance between the stimulating sites by the difference in latency time.

### 2.3. Histological techniques

Immediately after the recording of muscle action potential, all of the rats were sacrificed and perfused transacrdially with 150 *ml* normal saline followed by 300 ml 4% paraformaldehyde in 0.1 M phosphate buffer, pH 7.4. After perfusion, both intact and injured sides of their gastrocnemius muscles were dissected, harvested, and weighed while still wet using an electronic balance. A ratio of the gastrocnemius muscle of the injured side to the normal side was measured. The L4 spinal cord and the distal stump outside the nerve gap were quickly removed and post-fixed in the same fixative for 3 to 4 hours. Tissue samples were placed overnight in 30% sucrose for cry protection at 4°C, followed by embedding in optimal cutting temperature solution. Samples were the kept at -20°C until preparation of 18 μm sections was performed using a cryostat, with samples placed upon poly-L-lysine-coated slide. Immunohistochemistry of frozen sections was carried out using a two-step protocol according to the manufacturer’s instructions (Novolink Polymer Detection System, Novocastra). Briefly, frozen sections were required endogenous peroxidase activity was blocked with incubation of the slides in 0.3% H_2_O_2_, and nonspecific binding sites were blocked with Protein Block (RE7102; Novocastra). After serial incubation with rabbit-anti-CGRP polyclonal antibody 1:1000 (Calbiochem, Germany), Post Primary Block (RE7111; Novocastra), and secondary antibody (Novolink Polymer RE7112), the L4 spinal cord sections were developed in diaminobenzidine solution under a microscope and counterstained with haematoxylin. Similar protocols were applied in the sections from the distal stump except they were incubated with anti-rat CD68 (a pan-macrophage marker) 1:100 (AbD Serotec, Kidlington, UK). Sciatic nerve sections were taken from the middle regions of the regenerated nerve in the chamber. After the fixation, the nerve tissue was post-fixed in 0.5% osmium tetroxide, dehydrated, and embedded in Spurr’s resin. The tissue was then cut to 2-μm thickness by using a microtome (Leica EM UC6, Leica Biosystems, Mount Waverley, Australia) with a diamond knife, stained with toluidine blue.

### 2.4. Image analysis

All tissue samples were observed under an optical microscope (Olympus IX70; Olympus Optical Co, Ltd, Tokyo, Japan) with an image analyzer system (Image-Pro Lite; Media Cybernetics, Silver Spring, MD). CGRP-immunoreactivity (IR) in dorsal horn in the lumbar spinal cord was detected by immunohistochemistry as described previously. The immuno-products were confirmed positive-labelled if their density level was over five times background levels. Under a 400x magnification, the ratio of area occupied by positive CGRP-IR in dorsal horn ipsilateral to the injury following neurorrhaphy relative to the lumbar spinal cord was measured. The number of neural components in each nerve section was also counted. As counting the myelinated axons, at least 30 to 50% of the sciatic nerve section area randomly selected from each nerve specimen at a magnification of 400* was observed. Axon density was then obtained by dividing the axon counts by the total nerve areas. Similarly, the density of macrophage was determined by dividing the macrophage counts by the total nerve areas.

### 2.5. Statistical analyses

For the statistical analysis of immunohistochemically, morphometric, and electrophysiological measurements of regenerated nerves, data were collected by the same observer and expressed as mean ± standard deviation (SD), and comparisons between groups were made by the one-way analysis of variance using SAS 9.4 (SAS Institute Inc., Cary, NC, USA). The Tukey test was then used as a post hoc test for a multiple comparison. Statistical significance was set at *P* < 0.05.

## 3. Results

### 3.1. Regeneration across gaps within silicone rubber conduits

Gross examination of the silicone rubber chambers at 4 weeks revealed higher successful regeneration in groups with swimming that 60% (6 of 10), 50% (5 of 10), and 60% (6 of 10) in S10, S20, and S30, respectively. In comparison, only 30% (3 of 10) of the animals in the SC group exhibited a regenerated nerve cable across the 10-mm gap within the bridging conduits.

### 3.2. Nerve regeneration following swimming at various levels of exertion

**Fig. 2:**
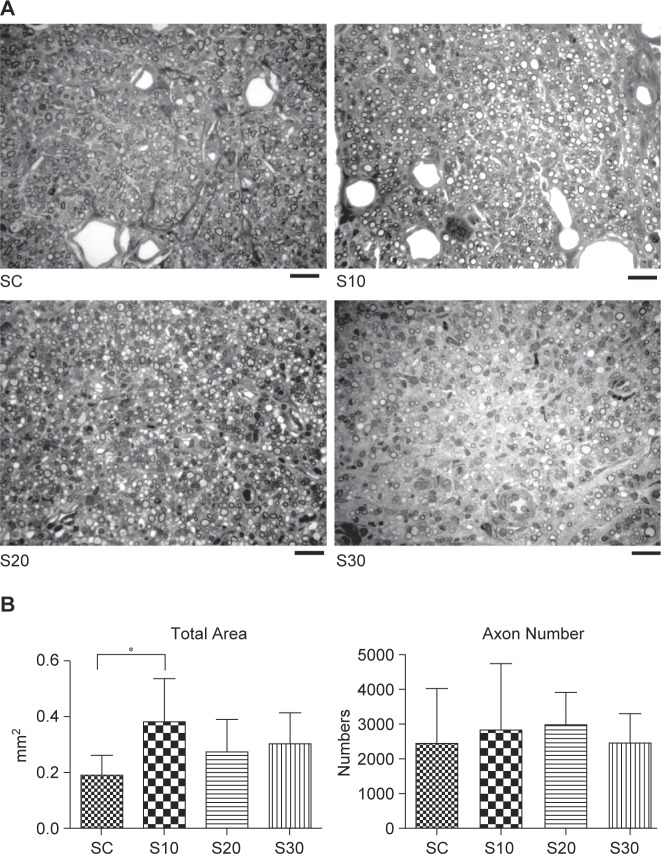
Effects of swimming excise on axon regeneration in rats after nerve injury surgery. (A) Histological micrographs of nerve tissue. (B) Quantitation of total nerve area and counts in regenerated sciatic nerve cross-sections is shown. The values represent means ± SD for each group. *Significant difference (*P* < 0.05) compared to SC group. Scale bar = 20 μm.

The representative photomicrographs of axon regeneration seen in the cross section of regenerated sciatic nerve, including total area and axon number, are shown in Fig. 2A. Total nerve regeneration area of the S10 group was significantly elevated to approximately two-fold more than that of the sedentary control (SC) group (*p* < 0.05). Although there were no statistically significant differences between the S20 and S30 groups, data showed that the total nerve regeneration area of these groups was also elevated by approximately 25% to 50% than that of the SC group. However, no significant differences were found between the groups for axon number because of the large variation in data (Fig. 2B).

### 3.3. Effects of swimming on electrophysiological function

**Fig. 3:**
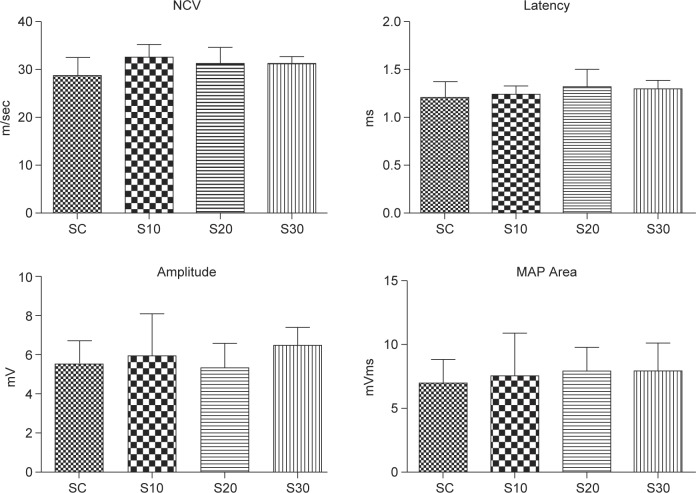
Effects of swimming excise on electrophysiological data, including NCV, latency, amplitude, and MAP area, of nerve function in rats after nerve injury surgery. The values represent means ± SD for each group.

The electrophysiological data demonstrated that nerve function, including NCV, latency, amplitude, and MAP area of the nerve post-injury did not differ significantly according to swimming regime (Fig. 3).

### 3.4. Effects of swimming on muscle weight

**Fig. 4:**
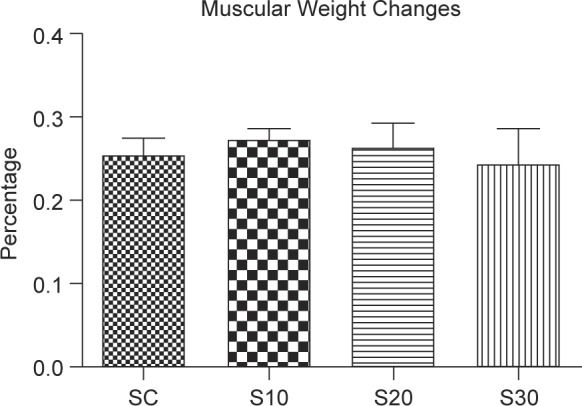
Effects of swimming excise on muscular weight changes in rats after nerve injury surgery. The values represent means ± SD for each group.

Muscle weight has been considered as a functional parameter, influencing nerve regeneration. The results showed that swimming did not affect muscle weight in the parallel limb of rats (Fig. 4).

### 3.5. Effects of swimming on macrophage infiltration

**Fig. 5:**
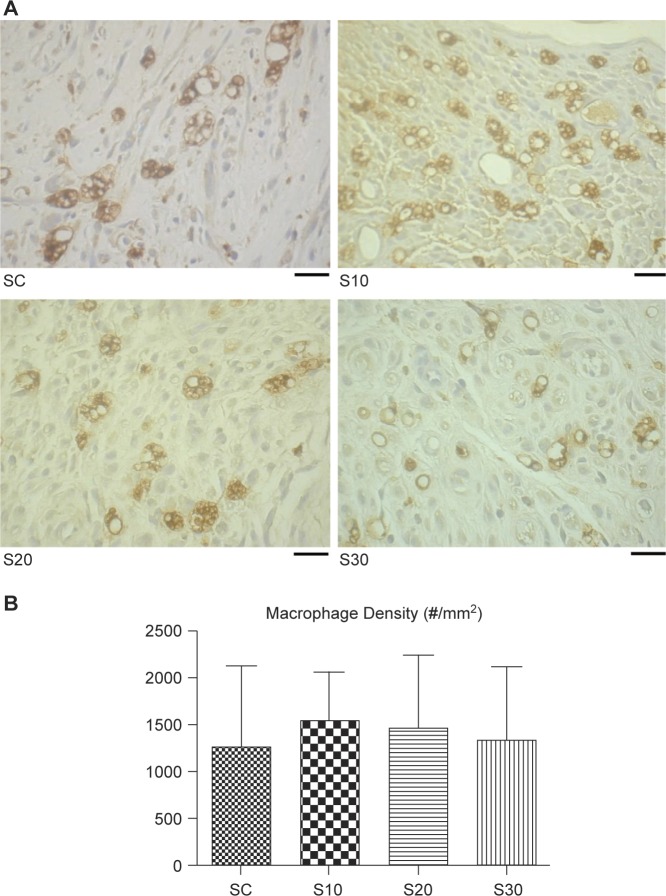
Effects of swimming excise on macrophage infiltration in rats after nerve injury surgery. (A) Histological micrographs of macrophage infiltration. (B) Quantitation of macrophage infiltration density. The values represent means ± SD for each group. Scale bar = 20 μm.

Post-injury macrophage infiltration revealed that continued damage and post-damaged clearance existed in the nerve injury lesion of the spinal cord, and the inflammation persisted in the postinjury lesion. Our data showed that macrophage morphology (Fig. 5A) and infiltration density (Fig. 5B) were not related to exercise regime.

### 3.6. Effects of swimming on CGRP expression

**Fig. 6:**
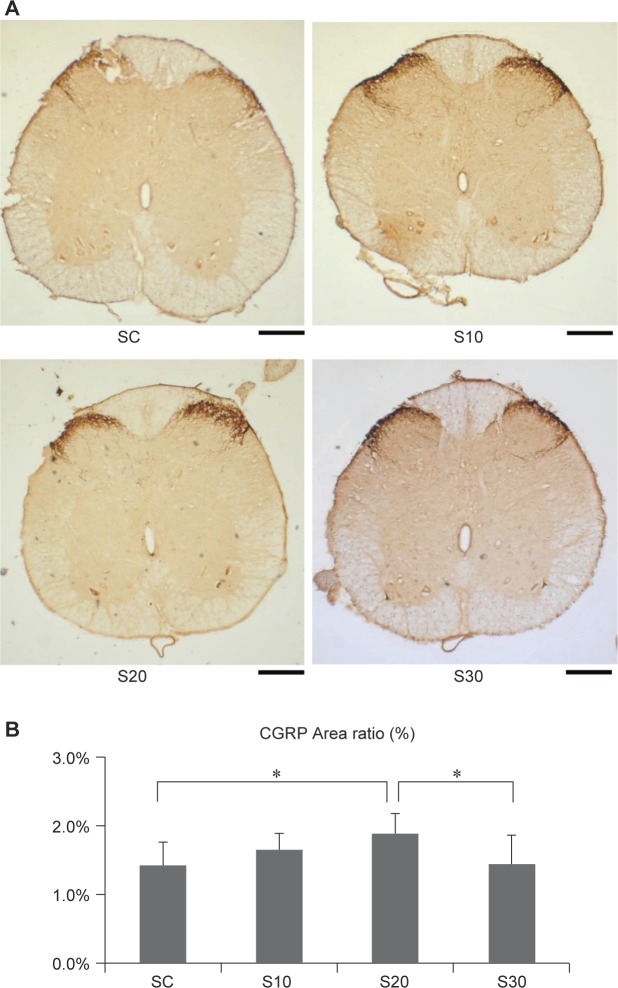
Effects of swimming excise on CGRP expression in rats after nerve injury surgery. (A) Histological micrographs of CGRP expression. The anatomic position of CGRP expression was separately accounted for the dorsal and ventral positions. The CGRP of dorsal horn showed the highest expression of whole horizontal view of spinal cord (B) Quantitation for the ratio of CGRP expression area. The values represent means ± SD for each group. *Significant difference (*P* < 0.05) compared to SC group. Scale bar = 200 μm.

The anatomic position of CGRP expression was measured separately for the dorsal and ventral positions. CGRP of the dorsal horn showed the highest expression in the whole horizontal view of spinal cord (Fig. 6A). The regenerative index CGRP data was significantly elevated in the S20 group compared to that in the SC group (*p* < 0.05). However, CGRP of the S10 and S30 groups was not significantly different (Fig. 6B). Since the CGRP could promote nerve regeneration after injury that the data suggest moderate exercise could accelerate nerve regeneration.

## 4. Discussion and conclusion

In the present study, we investigated the effects of various swimming exercise regimes on nerve regeneration in a rat sciatic nerve transection model. The results showed that moderate swimming rehabilitation therapy could significantly improve nerve regeneration in rats after acute nerve injury. In addition, CGRP might play an important role in swimming exercise-related nerve function and repair after axons have been severed.

One previous study showed that exercise types [10] and further alteration of treatment [11] may affect myopathic changes in the reinnervation period. However, swimming is a complex endurance exercise; short forced swimming exercise could elevate the mobility response alarm substance which were be a clue for notice the damage of forced exercises [12]. Thus, duration and exercise type would be more important factors in a swimming exercise model. Another approach suggested that heavy exercise regimes may reduce nerve regeneration [13], thus these approaches were existed inconsistency results of animal model. In this study, we clarified the effect of swimming exercise and duration on nerve regeneration using a nerve repair animal model for long distances.

Different exertion levels of swimming rehabilitation influence the interplay of nerve-associated cells, including macrophages and Schwann cells [14]. Moreover, individual differences, such as adaptive capacity, would also influence post-injury nerve regeneration. Sarikcioglu and Oguz investigated the relationship between exercise training and peripheral nerve regeneration after crush injury and showed that exercise is effective in the fourth regeneration week [15]. Ilha *et al.* also evaluated the effects of endurance, resistance, and a combination of both types of exercise training on hind limb motor function recovery and nerve regeneration after experimental sciatic nerve lesion in rats. They showed that endurance training improves sciatic nerve regeneration after an experimental traumatic injury and that resistance training or the combination of the two strategies may delay functional recovery and do not alter sciatic nerve fiber regeneration [16]. Magnuson *et al.* also suggested that force (load) and pattern generation (recruitment) are independent and may have to be managed together with respect to post-injury rehabilitation [17].

Macrophages are the most notable immune cells that play a key role in peripheral nervous system (PNS) injury and repair, and swimming might play a role in the modulation of inflammation of the target tissue. Successful PNS regeneration relies on both injured axons and non-neuronal cells, including Schwann cells and immune cells. Upon nerve injury, macrophages infiltrate the injury sites, where they not only contribute to Wallerian degeneration but are also influenced by the local microenvironment and are polarized to an anti-inflammatory phenotype (M2), contributing to axonal regeneration [18]. In this study, we could not analyze the effect of swimming on macrophage infiltration into the endoneurium following nerve injury in rats. Apart from their role in removing myelin debris from the degeneration process, the macrophages and their released interleukin (IL)-1 p were found in a previous study [19] to also stimulate the secretion of various growth factors in dissected nerve segments, which could exert neurotrophic effects on regenerating nerve fibers [20, 21]. The underlying mechanisms involved in moderate swimming could potentially accelerate the nerve regeneration process and promote neurotrophic factors, leading to enhancement of the regenerative response, and this needs to be further investigated.

CGRP is produced in both peripheral and central neurons and is a potent peptide vasodilator that can function in the transmission of pain [9]. In a spinal cord injury, CGRP is derived from motor neurons and plays a role in nerve regeneration after injury. Conversely, CGRP is derived from dorsal root ganglion when synthesized in the dorsal horn of the spinal cord and may be involved in transmission of post-injury pain [9]. Previous studies showed that CGRP stimulates specific progenitor cells, which secrete an insulin-like growth factor, leading to regeneration [22], and CGRP represses specific immune cells, such as T lymphocytes, *via* repression of IL-2 and nuclear factor-kB [23].

Moreover, CGRP could be a bio-signature for the surveillance of the basal and dorsal root CGRP enhancement, which might reflect the physiological status of the synaptic connections in the spinal dorsal horn [24]. In the present study, swimming influenced the systemic homeostasis of low gravity rehabilitation after sciatic nerve post-injury, such as the reduction of immune responses and toleration of the pain [25], which would affect the behavioral properties for improvement of swimming rehabilitation in rats.

Limb stretching facilitates post-injury functional nerve recovery during the rehabilitation period in rats [26]. Previous studies have shown that exercise interventions are effective for nerve injury [27]. Gutmann and Jakoubek reported that swimming increased axonal growth following sciatic nerve damage and that exercises such as, running on a treadmill increased reinnervation, axonal elongation, and sprouting [28]. More recently, Kim *et al.* found no difference between weight bearing (treadmill) and non-weight-bearing exercises following sciatic nerve damage, and both exercises accelerated the recovery process [29]. These results are consistent with the results of our study, showing that moderate swimming was effective in nerve recovery. However, we noticed that a heavy swimming regime for long duration and frequency could have an adverse effect during rehabilitation, especially in the post-injury rehabilitation with long distance of traumatic injury. On the other hand, another study reported that limb immobilization post-injury could improve rehabilitation and alter functional recovery [30].

In this study, we investigated the effects of different swimming regimes on nerve regeneration in a rat sciatic nerve transection model, and the evidence supported that moderate exercise adequately improved nerve regeneration. We demonstrated that moderate swimming could increase CGRP expression in the dorsal horn and was associated with higher successful percentages of nerve regeneration across the 10-mm gaps and elevated axon numbers in larger regenerated nerves in the acute phase of postinjury nerve regeneration. These results should inspire new studies of physiotherapeutic practice for related human treatment.

## Specific author contributions

C-F Liao, T-D Way, and Y-S Chen were responsible for the study design, coordination, and drafting of the manuscript. C-F Liao and T-Y Yang collected data and performed analysis. T-D Way, C-H Yao, Y-H Chen, and Y-S Chen provided guidance and reviewed the manuscript. C-F Liao, C-H Yao, and T-D Way contributed equally to this work. All authors collaborated in writing the final version of the manuscript. All authors read and approved the final manuscript.

## Conflicts of Interest Statement

The authors report no conflicts of interest or financial interests associated with this work, both collectively and individually.
